# Metabolism, inflammation, and cardiovascular diseases from basic research to clinical practice

**DOI:** 10.1097/CP9.0000000000000037

**Published:** 2023-03-27

**Authors:** Zihang Huang, Aijun Sun

**Affiliations:** 1 Department of Cardiology, Zhongshan Hospital, Fudan University, Shanghai 200032, China.; 2 Shanghai Institute of Cardiovascular Diseases, Shanghai 200032, China.; 3 Institute of Biomedical Sciences, Fudan University, Shanghai 200433, China.

Cardiovascular diseases (CVDs) affect more than 500 million people worldwide and are associated with high mortality and morbidity^[1]^. The heart uses a variety of substrates as energy source, including fatty acid, glucose, lactate, ketone body, and branched-chain amino acid^[2]^. Metabolic changes that occur in metabolic diseases have been implicated in the development and patient prognosis of most CVDs. Metabolic changes also promote systemic and cardiac inflammation, thus forming a vicious cycle to exaggerate myocardial injury^[3]^. Reciprocally, immune cells could interact with cardiomyocytes to trigger a variety of metabolic reprogramming^[4]^. The crosstalk between cardiac metabolism and inflammation is highly complex^[4]^. Accordingly, developing preventive and treatment strategies using metabolism and inflammation for CVDs is promising but highly challenging. This special issue of Cardiology Plus on “Metabolism, Inflammation and Cardiovascular Diseases-from Basic Research to Clinical Practice” showcases the latest evidence and creative opinions on the relationship between inflammation and metabolism within the context of CVDs. We believe that this discussion will facilitate mechanistic studies that eventually may lead to novel therapeutic options.

Metabolic shift in cardiomyocytes after insults is a self-defensive mechanism against detrimental stimuli. A combination of single-cell RNA-sequencing and spatial transcriptomics analysis allows investigation of the changes at the single cell level, and is helpful in understanding the basis of global changes^[5]^. Using spatial single-cell transcriptomics, Dr. Shen, Prof. Tang (Augusta University, USA) and his colleagues^[6]^ demonstrated severe metabolic shift in cardiomyocytes that survived myocardial infarction in human subjects in their study. The changes included downregulation of fatty acid oxidation, mitochondrial oxidative phosphorylation (OXPHOS) and tricarboxylic acid cycle (TCA) activities, and a concomitant increase in the biosynthesis of fatty acids; these changes are known to protect membrane integrity and inhibit ROS generation upon inflammatory injury. The surviving cardiomyocytes displayed higher purine and pyrimidine metabolism, indicating a potential DNA repairing ability against oxidative stress. Intriguingly, glycolysis was also inhibited in surviving cardiomyocytes. Further metabolomics may help understand how surviving cardiomyocytes decreased energy supply due to lower utilization of most energy substrates. In addition to cardiomyocytes, the metabolic stability of vascular cells was also affected by circulating metabolites under pathological stimuli. Cardiovascular calcification is closely associated with inorganic ion metabolic disorder of vascular smooth muscle cells (VSMCs). In a research conducted by Prof. Geng (University of Texas, USA)^[7]^, deficiency of galactosyltransferase 1-associating protein (UBE2Q1), an E2 ubiquitin-conjugating enzyme, exacerbated VSMCs calcification under high concentration of inorganic phosphate. Mechanistically, UBE2Q1 induced polyubiquitinated degradation of Runt-related transcription factor 2 (RUNX2), a critical transcriptional factor for osteogenesis and macrophage activation, thus protecting aortic tissues from calcification. As the central mediator of chronic or acute inflammation development, macrophages have long been considered as significant targets against cardiovascular injuries. Prof. Li (The University of Texas, USA)^[8]^ summarizes the impact of macrophages on CVDs via inflammasome activation in their study. Inflammasomes are activated in response to exogeneous or endogenous danger stimuli, thus “alarming” innate immune cells to transform into a “proinflammatory status.” Inhibiting NOD-like receptor thermal protein domain-associated protein 3 (NLRP3) inflammasome, thus, may represent a promising approach to manipulate macrophage polarization and ultimately improve the survival of cardiomyocytes under stress. Since inflammasome could be activated by cholesterol crystals and fatty acid burden, regulating fatty acid metabolism in noncardiomyocytes is a promising approach in the treatment of CVDs. The exploration conducted by Prof. Pei (Southwest Medical University) *et al*^[9]^. showed that serum uric acid could be used as a biomarker for estimating the openness of coronary collateral circulation and predicting the prognosis of patients with coronary chronic total occlusion (CTO). Specifically, they studied 94 patients undergoing coronary angiography for coronary CTO, and demonstrated a negative association between the serum uric acid level and coronary collateral openness. Prof. Chen (National Yang-Ming University, Taipei, Taiwan)^[10]^ reported an association between the higher level of circulating endothelial progenitor cells (EPCs) with the risk of adverse cardiovascular clinical outcomes in 1,099 patients undergoing nonemergent coronary angiography excluding those with peripheral artery disease. Further studies are needed to validate such a finding and to investigate why a biomarker of vascular repairing is associated with adverse outcomes. Moreover, Prof. Wu (Xi’an Jiaotong University, China) *et al*^[11]^. reported that in comparison to statin treatment alone, statin plus evolocumab could decrease the lipoprotein(a) level in patients with acute MI, indicating the importance of regulating fatty acid metabolism even in nonarteriosclerosis CVDs.

Cardiovascular injury caused by inflammation is augmented in the setting of metabolic diseases^[12]^. Accordingly, correcting metabolic abnormality could be useful in the treatment of CVDs not typically associated with metabolic diseases, for example, myocardial infarction. A key barrier in developing such strategies is the fact that metabolic shift varies substantially among different cells. Investigating the metabolic alterations at the single cell level rather than in whole tissue could provide more information. Studies of metabolic alterations in cardiomyocytes versus other types of cells, including fibroblasts, endothelial cells, mast cells, and smooth muscle cells, are also needed. Currently, immune cells are grouped into subpopulations based on biomarkers. A novel classification of cardiac immune cells based on metabolic status would be helpful. Last but not least, the efficacy and safety of newly developed treatment strategies must be ultimately tested in patients.

## CONFLICTS OF INTEREST STATEMENT

Aijun Sun is an Editorial Board member of *Cardiology Plus*.
